# LPS immune challenge reduces arcuate nucleus TSHR and CART mRNA and elevates plasma CART peptides

**DOI:** 10.1186/s12868-019-0539-z

**Published:** 2019-12-11

**Authors:** Jonathan R. Burgos, Britt-Marie Iresjö, Linda Olsson, Ulrika Smedh

**Affiliations:** 1 Department of Surgery, Institute of Clinical Sciences, Sahlgrenska Academy, Gothenburg, Sweden; 2 Department of Surgery, Sahlgrenska University Hospital, Region Västra Götaland 41345 Gothenburg, Sweden

**Keywords:** Arcuate nucleus, Paraventricular nucleus, Cocaine- and amphetamine-regulated transcript, Thyrotropin, Lipopolysaccharide

## Abstract

**Background:**

The aim was to examine the impact of lipopolysaccharide-induced systemic inflammation on expression of mRNA for cocaine- and amphetamine-regulated transcript (CART) and the thyrotropin receptor (TSHR) and its ligands in CNS areas of relevance for feeding controls and metabolism. Lipopolysaccharide effects on plasma levels of TSH and CART peptides were also examined.

**Methods:**

Lipopolysaccharide (150–200 μg/mouse) was injected in C57BL/6J mice and tissue and plasma samples taken after 24 h. To establish if plasma increase in CART peptide levels were prostanoid dependent, indomethacin was given via the drinking water beginning 48 h prior to LPS. We evaluated mRNA expression for CART, TSHR, TSHβ, and thyrostimulin in brain and pituitary extracts. Plasma levels of TSH, CARTp, and serum amyloid P component were analyzed by ELISA.

**Results:**

Lipopolysaccharide suppressed TSHR mRNA expression in the arcuate nucleus and the pituitary. CART mRNA expression was reduced in the arcuate nucleus but elevated in the pituitary of mice treated with Lipopolysaccharide, whereas plasma TSH remained unchanged. Plasma CART peptide concentration increased after LPS treatment in a prostanoid-independent manner, and CART peptide levels correlated positively to degree of inflammation.

**Conclusions:**

Our findings suggest that central and peripheral CART is affected by acute inflammation. Considering the role of the arcuate nucleus in feeding controls, our data highlight TSHR and CART as putative neuroendocrine signaling components that respond to inflammation, perhaps to maintain weight and metabolic homeostasis during states of disease.

## Background

The impact of acute inflammation on the central and peripheral expressions of cocaine- and amphetamine-regulated transcript (CART), the thyrotropin receptor (TSHR) and its ligands, TSH and thyrostimulin, in the mouse is investigated in this study. We recently described that central expressions of TSHR, CART and plasma CART peptides (CARTp) are altered in a pro-inflammatory tumor model that displays high circulating levels of PGE2 [1]. Here, we extend our investigations to include possible causal effects of acute inflammation and role of prostanoids. Our rationale for selecting these signaling components was that these may affect some physiological features that are also altered during inflammation. Induction of acute inflammation results in alterations in metabolic function, increase in core temperature, and anorexia [2–4]. Lipopolysaccharide (LPS) is a commonly used experimental tool to induce such acute inflammation. Previous investigations aimed at determining the neural networks activated by acute inflammation found that Fos-like immunoreactivity was elevated in autonomic regulatory brain regions including the paraventricular (PVN) and arcuate (ARC) nuclei, and the nucleus of the solitary tract (NTS) after LPS [5, 6]. These nuclei participate in regulating host metabolic processes, feeding behavior, and visceral signaling.

In the rat, LPS treatment impacts hypothalamic mRNA expression for a variety of neuropeptides including CART [7, 8]. CART and its peptides (CARTp) are endogenous substances that influence feeding, autonomic functions, motor behavior, and core temperature [9–12]. In rats, LPS increases CART mRNA in the ARC and in neurons of the PVN that project to the dorsal vagal complex of the brainstem [7, 8, 13]. Since investigations of acute inflammation on CART expression within the CNS have mostly been performed in the rat, it has yet to be determined whether similar neuroendocrine responses also occur in the mouse.

The location of TSH and its receptor in peripheral tissues is well established. However, both genes and corresponding proteins are also present in the CNS including the hypothalamus [14–19]. TSHR protein is present in structures associated with feeding regulation, including the hypothalamic arcuate nucleus [20]. Moreover, there is support for possible connections between some TSH mechanisms and CART at the CNS level [21–26]. In addition, intracerebroventricular (i.c.v) injections of TSH were reported to reduce feeding and affect thermoregulation in rats [27, 28]. Besides TSH, thyrostimulin is proposed as an acutely inducible TSHR agonist which is present in the CNS and that may impact TSH feedback mechanisms [29–31]. It has recently been reported that acute LPS reduces hypothalamic and pituitary expression of the TSHR mRNA in wildtype but not thyrostimulin-knockout mice [19]. In addition, LPS significantly increased expression of thyrostimulin mRNA in the hypothalamus and pituitary at 24 h after exposure [19]. Here we investigate the effect of acute inflammation on central and peripheral expression of CART, TSH, TSHR and thyrostimulin in the mouse. The present study thus had three basic aims: first, investigate LPS-induced changes in TSH, TSHR, CART, and thyrostimulin mRNA expression within the brainstem, ARC, PVN, and pituitary. Second, to examine whether such changes in mRNA expression are paralleled by alterations in circulating TSH and/or CARTp levels; and lastly we tested the hypothesis that an LPS-induced increase in plasma CARTp levels is cyclooxygenase dependent.

## Methods

### Animals and experimental design

Adult female mice weighing 18–23 g were used (C57Bl/6JBomTac; Taconic, Denmark). Animals were group-housed under controlled ambient conditions: lights on 07:00–19:00 h; temperature 21 ± 1 °C; relative humidity 45–55%. The housing cages were kept in close proximity for synchronization of oestrus cycles among mice for more than 10 days before the start of the study. Animals had free access to standard rodent maintenance chow and drinking water throughout the study. All study protocols were approved by the Gothenburg Regional Animal Ethics Committee and all care and use was in accordance with national and institutional guidelines. The experiment was performed in an animal testing laboratory.

**Fig. 1:**
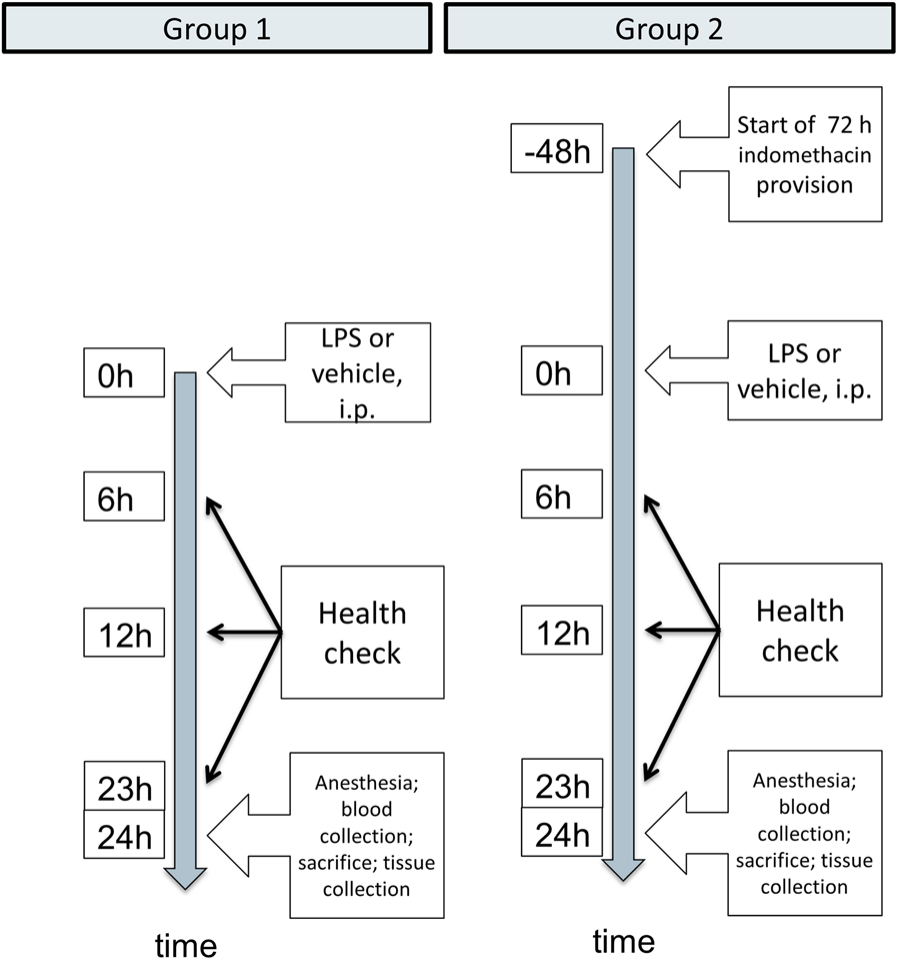
Timeline of the study

The animals were randomly assigned to two groups of mice. An experimental timeline is shown in Fig. 1. The first group was given single intraperitoneal injections (i.p.) of 200 µg LPS (n = 5) (*Salmonella enterica*, serotype Enteritidis; Sigma-Aldrich, Germany) dissolved in 0.9% saline solution, and weight-matched controls n = 6) were injected with an equivalent volume (0.2 mL) of the saline vehicle. As part of the ethical protocol, the animals were checked for signs of illness (lethargy, piloerection, lack of grooming) at 6 h and 12 h after injection, and 1 h prior to sacrifice as well.

In the second group, LPS mice and weight-matched controls were injected as above. However, a reduced LPS dose (150 µg/mouse) was used since one LPS-treated animal from the first group showed signs of illness and died just prior to sample collection, and could thus not be included. Eight LPS mice and six saline-injected mice received indomethacin (INDO) (6.7 µg/mL, 0.13% v/v ethanol; Sigma-Aldrich, Germany) in the drinking water beginning two days prior to i.p. injections. Since INDO was dissolved in ethanol, corresponding control mice (eight LPS mice and six saline-injected mice) subsequently received drinking water with ethanol vehicle added (0.13% v/v). The INDO concentration was selected to yield a daily dose of approximately 1 mg/kg mouse [32]. Respective drinking water mixtures were replenished once daily for a total of 3 days.

Gene expression analysis and analysis of plasma TSH levels were performed using samples collected from the first experimental group (LPS n = 5, controls n = 6). Body weights as well as plasma CARTp and SAP levels were collected from all mice (control mice n = 12; LPS n = 13; INDO/control n = 6; INDO/LPS n = 8).

### Tissue collection

Mice were anesthetized with a mixture of xylazine (5 mg/kg) and ketamine (100 mg/kg) i.p. in a volume of 0.1 mL/mouse. EDTA-blood was collected by cardiac puncture, placed on ice and then centrifuged for 10 min at 2000×*g*, at 4 ℃. The plasma was stored at − 80 ℃ until analysis. Blood and tissue sampling took place 23 ± 1 h after i.p. injections.

Brains and pituitaries were collected for mRNA expression analysis. Coronal transections of the brain were done in a standardized way, using a brain mold slicer and scalpel blades. The prepared slices from which the respective hypothalamic regions were subsequently dissected approximately represented the following levels according to the atlas of Franklin and Paxinos: − 0.5 mm to − 1.1 mm of the Bregma for the PVN; and − 1.1 mm to − 2.4 mm dorsal of the Bregma for the ARC. From the segment preparations, two areas were micro dissected in a standardized way using two 1 mm biopsy punches. The ARC was defined as the tissue around and below the lower third ventricle. The PVN was defined as the tissue surrounding the dorsal two-thirds of the third ventricle. Finally, the brainstem and the pituitary were dissected free. All tissues were immediately placed in RNAlater solution and immersed for 24 h at 4 **°**C according to manufacturer’s instructions (Ambion, Life Technologies) and then stored at − 20 **°**C until processing.

### Real time PCR

Tissues were homogenized with a rotor/stator homogenizer in QIAzol lysis reagent supplied in the RNeasy Lipid Tissue Mini Kit (Qiagen AB, Sweden). Total RNA was extracted according to the manufacturer’s protocol including the DNA digestion step. RNA concentration was determined using a Nanodrop ND-1000 instrument, and RNA integrity was established using a Bioanalyzer 2100 and RNA 6000 Nano kit (Agilent Technologies, Sweden). The RNA integrity numbers were above 7.0 for all tissue extracts.

Complementary DNA was synthesized from total RNA using the Advantage ^®^ RT for PCR Kit with oligo d(T)-primer (Clontech Laboratories, Inc., USA) according to manufacturer’s protocol. Brainstem, pituitary, and PVN cDNA was synthesized from 0.5 µg RNA. ARC samples had lower total RNA yield and therefore 0.2 µg RNA was used for cDNA synthesis.

Real time PCR was performed in a LightCycler 1.5 instrument with FastStart DNA Master^PLUS^ SYBR Green I (Roche Diagnostics GmbH, Germany) or QuantiTect SYBR Green PCR (Qiagen) master mixes. Two microliters of cDNA, 2 μL prepared primers, 4 μL SYBR Green I master mix, and 12 μL PCR-grade water were combined to yield 20 μL reactions for TSH, TSHR, CART, and GAPDH mRNA expression assays. Thyrostimulin reactions consisted of 5 μL cDNA, 2 μL prepared primers, 10 μL QuantiTect SYBR Green master mix, and 3 μL PCR-grade water. Commercially available QuantiTect Primer Assays (Qiagen) for TSHβ (*Tshb*, QT00135303), TSHR (*Tshr*, QT00136955), thyrostimulin (*Gpb5*, QT00154770), and CART (*Cartpt*, QT00130396) were used. The reaction capillaries underwent 40–50 thermocycles with temperatures set according to respective master mix protocols. GAPDH (*Gapdh*, QT001658692) was used as the housekeeping gene [33, 34]. Expression of *Gapdh* was similar across groups. Only one PCR product was detected in each analysis. Quantitative results were produced using the relative standard curve method. The efficiency values for the respective assays were: *Tshb* (QT00135303) 85%, r = − 1.00, *Tshr* (QT00136955) 85%, r = − 0.99, *Cartpt* (QT00130396), 85%, r = − 1.00 and *Gapdh* 85%, r = − 1.00, respectively. Due to low transcript levels, a reliable tissue standard curve for *Gbp5* could not be obtained in brain tissue. Thus, thyrostimulin expression was semi-quantitatively assessed by comparing rank scores derived from threshold cycle (Ct) values.

### Plasma assays

Plasma concentrations of TSH, CARTp, and serum amyloid P component (SAP) were determined by ELISA. Plasma aliquots were thawed on ice, vortex mixed, and centrifuged prior to sample preparation with respective kit reagents (Kamiya Biomedical Company, USA; RayBiotech, Inc., USA; R&D Systems Europe Ltd, UK). The assays were performed on individual samples in duplicate according to manufacturer-supplied protocols. Due to limited sample volume from some individuals in the second group of animals (indomethacin experiment; Table 1), some plasma samples were pooled pairwise in equal volumes and singularly assayed for CARTp concentration. Individual SAP concentrations were used for statistical analyses except for the regression analysis, where average values for the corresponding SAP measurements for each pooled CARTp pair in question were calculated.

**Table 1:** Group average plasma concentrations (± SEM) of TSH, CARTp and SAP, in LPS and vehicle-treated controls, with and without indomethacin

	Control	LPS	P	INDO/control	INDO/LPS	P
TSH pg/mL	200 ± 61 (n = 6)	181 ± 77 (n = 5)	ns	Nd	Nd	**–**
CARTp pg/mL	647 ± 125 (n = 9)	1428 ± 356 (n = 8)	< 0.05	671 ± 130 (n = 3)	4855 ± 1688 (n = 4)	< 0.01
SAP μg/mL	3.7 ± 0.5 (n = 12)	12.1 ± 1.6 (n = 13)	< 0.001	4.9 ± 0.4 (n = 6)	44.8 ± 4.4 (n = 8)	< 0.001

Student’s *t* test was used for comparing LPS-injected animals versus the respective control group

*n* sample size, *Nd* not done, *ns* not significant

P < 0.05 was considered significant

### Statistical analysis

Data are presented as mean ± SEM. Student’s *t* test was used to compare differences in weight change in response to LPS versus vehicle. Mann–Whitney U-test was used for statistical comparisons of differences in mRNA expression. Plasma concentration data was log-transformed before statistical analyses, since normal distribution could not be assumed. After this, pair-wise comparisons in plasma concentrations in response to LPS treatment or vehicle, was done for each respective protein. Spearman’s correlation test was used for correlation analysis. P-values less than 0.05 were regarded as statistically significant. Statistical analyses were performed using SPSS Statistics version 20 (IBM Corporation, USA).

## Results

**Fig. 2:**
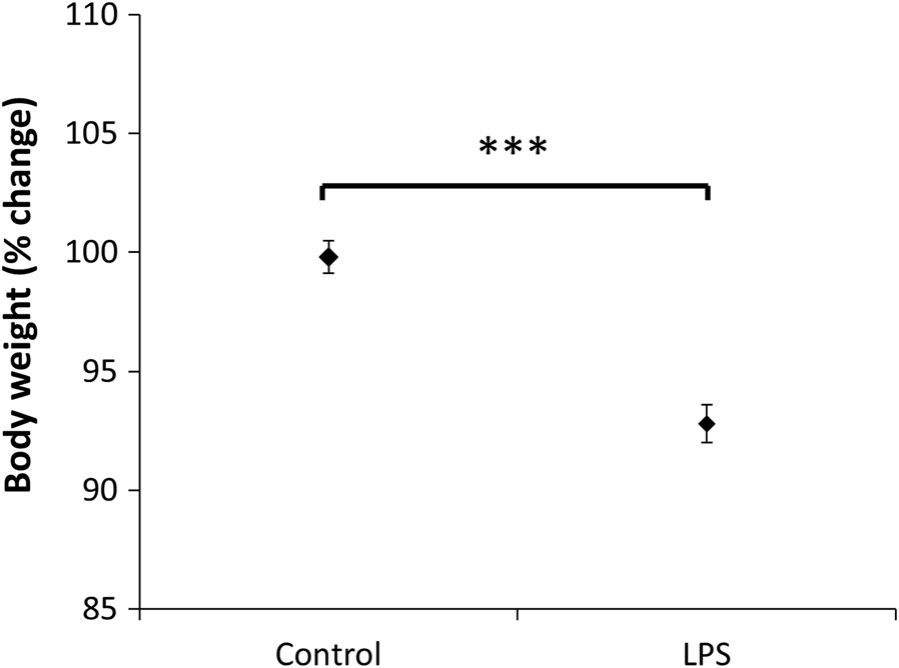
Body weight change (%) in vehicle-treated (n = 12), and in LPS-treated (n = 13) animals at 24 h. Data are expressed as mean ± SEM. Student’s *t* test was used for data analysis. (***P < 0.001)

LPS-treated mice displayed a significant weight loss while control animals remained weight stable after 24 h (P < 0.001, Fig. 2). In contrast to the controls, they also displayed signs of illness-related behavior (lethargy and lack of grooming) during the final 6 h before sacrifice. There was no difference in water intake between groups. The magnitude of the weight loss did not differ between the LPS doses administered (P = 0.997). Indomethacin did not protect against LPS-associated weight loss (P = 0.73). However, we observed that indomethacin-treated LPS mice exhibited normal in-cage behavior compared to control LPS mice, which all showed signs associated with sickness behavior at 18–24 h after treatment.

### Relative mRNA expression

**Fig. 3:**
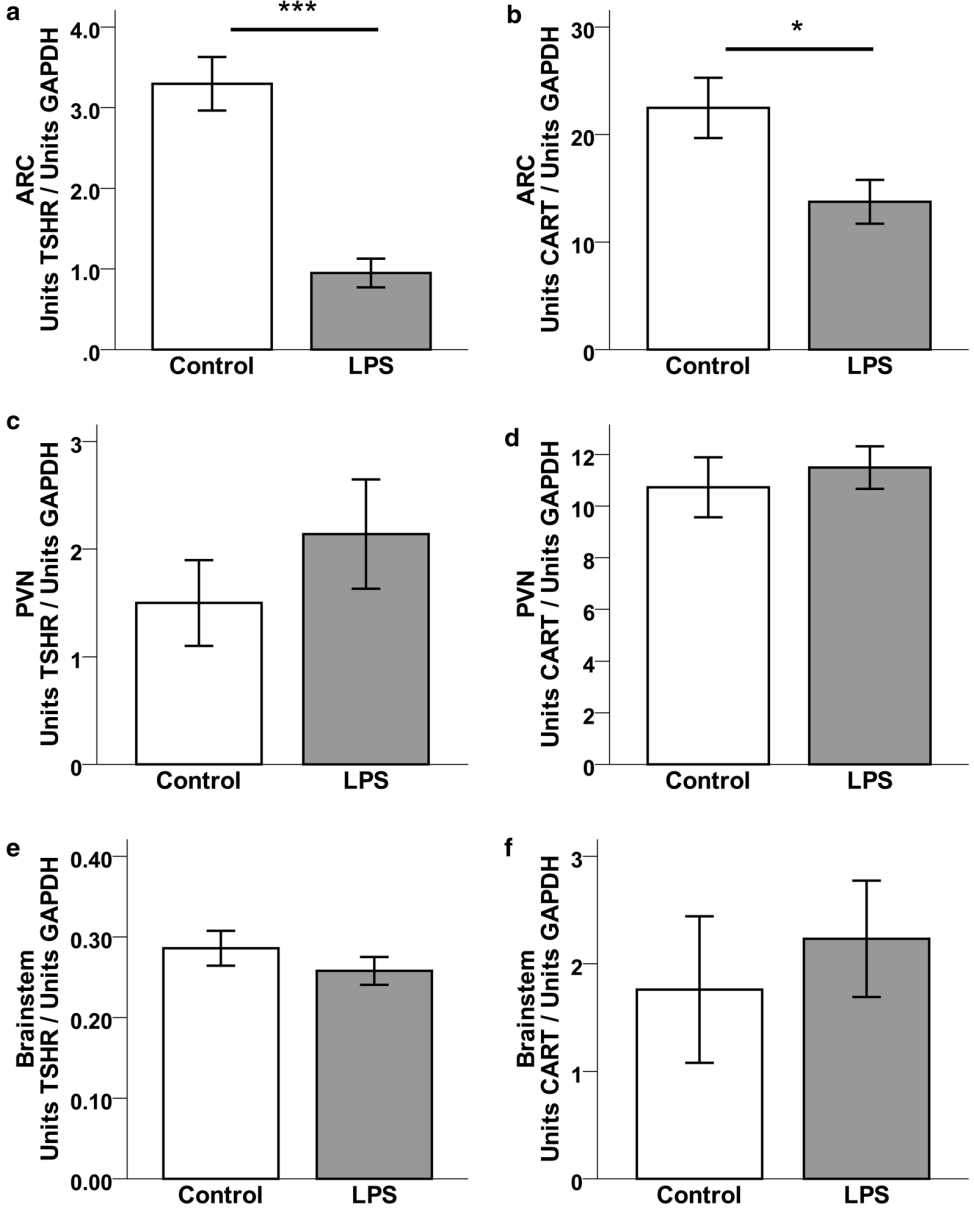
Relative mRNA expression (mean ± SEM units/units GAPDH) for TSHR (**a**, **c**, **e**) and CART (**b**, **d**, **f**) in the ARC, PVN, and brainstem of LPS-treated (n = 5) or control (n = 6) mice. Two-tailed Mann–Whitney U-test was used for data analysis. (*P < 0.05, ***P < 0.001)

**Fig. 4:**
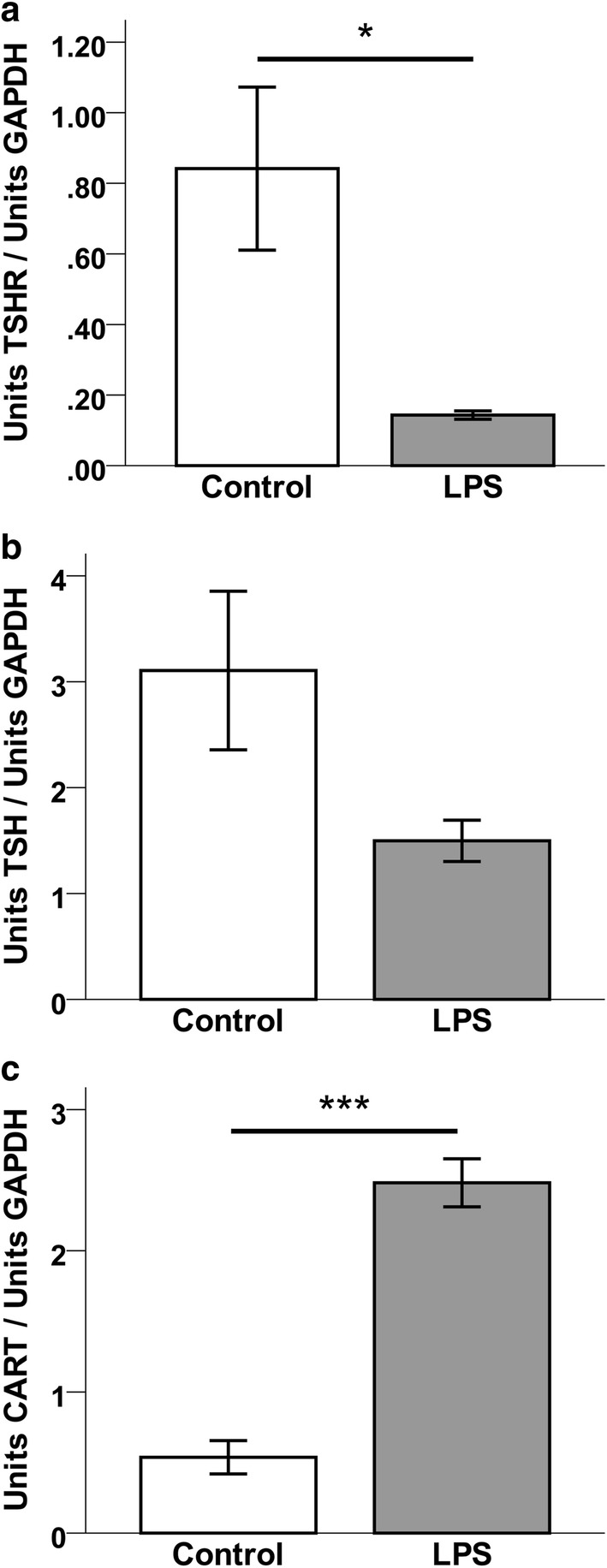
Relative mRNA expression (mean ± SEM units/units GAPDH) for **a** TSHR, **b** TSHβ, and **c** CART in the pituitary of LPS-treated (n = 5) or control (n = 6) mice. Two-tailed Mann–Whitney U-test was used for data analysis. (*P < 0.05, ***P < 0.001)

TSHR (Fig. 3a; P < 0.001) and CART (Fig. 3b; P < 0.05) mRNA levels were significantly lower in the hypothalamic ARC among LPS mice compared to controls. Expression in the PVN and brainstem did not differ between LPS and control mice with respect to TSHR and CART mRNA. TSHR mRNA was lower (P < 0.05) while CART mRNA was higher (P < 0.001) in the pituitaries of the LPS group compared to controls (Fig. 4).

TSHβ and thyrostimulin mRNA expression were detectable only in pituitary tissues. On average, there was a more than 50% reduction in TSHβ mRNA after LPS, however, this was not fully statistically significant (Fig. 4b; P = 0.09). All five mice receiving LPS had a thyrostimulin PCR product with an average threshold of 32.78 ± 0.39 cycles. Thyrostimulin transcript was detected in four of the six control mice (Ct = 35.50 ± 0.51 cycles, n = 4). The two control samples where thyrostimulin transcript was not detected were assigned Ct values of 45 cycles—the maximum number of reaction cycles—and a Mann–Whitney U test was performed. Although thyrostimulin mRNA levels were overall low, the distribution of Ct values between LPS mice and controls differed significantly. This indicates that pituitary thyrostimulin mRNA expression is increased in mice in response to LPS (P < 0.01).

### Plasma CARTp, TSH and SAP

**Fig. 5:**
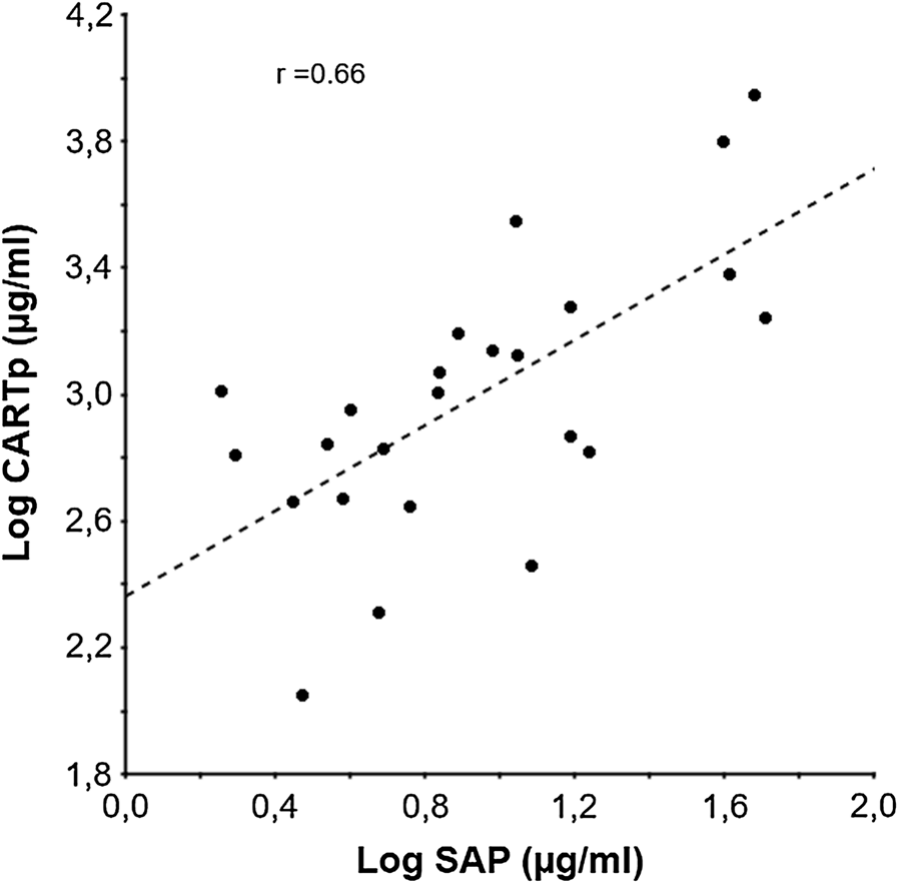
Linear correlation (r = 0.663, P < 0.0001) between SAP and CARTp plasma levels in mice (n = 24). For data analysis, Pearson’s correlation test was used

CARTp was significantly higher in LPS-treated mice compared to control mice (Table 1; P < 0.05). INDO treatment failed to inhibit the elevation of plasma CARTp (Table 1; P < 0.01). Plasma SAP concentration increased in response to LPS (Table 1; P < 0.001). In addition, subsequent Pearson’s correlation test revealed that CARTp and SAP plasma concentrations positively correlated (Fig. 5; r = 0.663, P < 0.0001, n = 24). Plasma TSH levels did not differ between LPS and control mice (Table 1; P = 0.77).

## Discussion

In the present study we explored the central and peripheral responses to LPS-induced inflammation with respect to CART, TSH, TSHR, and thyrostimulin in the mouse. LPS treatment led to a reduction of CART and TSHR mRNA expression in the ARC (Fig. 3a, b) but had no effect on mRNA expression in the PVN or the brainstem (Fig. 3c–f). Our current data are consistent with a previous report where there was a simultaneous elevation of pituitary thyrostimulin and depression of TSHR gene expression in the ARC 24 h after LPS treatment [19]. Pituitary TSHβ mRNA expression had a tendency to decrease in response to LPS (Fig. 4b). Taken together, these findings support the suggestion that hypothalamic and pituitary TSHR expression is down-regulated during states of acute inflammation [29–31, 35]. Furthermore, the present data indicates that while TSHR mRNA is indeed expressed in the mouse brainstem, these levels were not altered by acute systemic inflammation.

Our current CART mRNA expression data suggest that the transcript is differently expressed in the hypothalamus and the pituitary during states of acute inflammation. We found CART expression to be lowered in the ARC of immune-challenged mice (Fig. 3b). This finding contrasts with studies in the rat where LPS is associated with elevated CART mRNA expression in the ARC [7, 8]. Whereas this could be due to species differences, we cannot exclude that these contrasting results may be due to some variations in experimental design between studies. Feeding state and time of day influence basal hypothalamic CART expression [9, 36], and it cannot be excluded that the duration or degree of inflammation may also have an impact. With regard to LPS dose, direct comparisons are difficult to make, since the degree of the inflammatory response depends on the LPS source and batch, rather than the doses administered. Notably, a similar suppression of CART mRNA expression was seen in the hypothalamus of mice carrying a pro-inflammatory, PGE2-producing tumor [1]. Together with the present results, this supports the suggestion that hypothalamic CART mRNA is suppressed in response to inflammatory signaling in the mouse.

CART mRNA expression has previously been reported in the anterior pituitary [23, 37, 38]. The relative expression of CART mRNA extracted from whole mouse pituitaries was higher following LPS injections in the present study (Fig. 4c). The inverse directionality of CART mRNA expression in the hypothalamus versus the pituitary and plasma CARTp increase may support a two-compartment system response (central vs. peripheral) to acute endotoxemia. There is, as yet, little known about peripheral CARTp actions in healthy and diseased states. Our findings in conjunction with previous reports of an overlap between CARTp and pituitary hormone release may further implicate CARTp as a modulator for pituitary hormone secretion during states of acute systemic inflammation or stress, and it may perhaps play a role in non-thyroidal illness [1, 21, 23, 25, 38–40]. In this study we showed that LPS induces a robust increase in plasma CARTp levels (Table 1). The role for peripheral CARTp is not fully known; in contrast to central CARTp, acute peripheral administration of CARTp does for example, not affect food intake [41] or gastrointestinal function [42]. It is thus less likely that circulating CARTp, in physiological concentrations, exerts central effects by passing the blood brain barrier. Although the specific source for CARTp in the circulation is not known, our present results do suggest a possible role for plasma CARTp in the inflammatory response. Since a similar elevation of plasma CARTp was seen in mice bearing PGE2-producing tumors [1], we raised the hypothesis that the plasma CARTp elevation in response to LPS was prostanoid induced. As expected, INDO prevented apparent sickness behavior in LPS-treated mice [43], but it did not affect the plasma CARTp response (Table 1). We also found a positive correlation of plasma CARTp and SAP levels (Fig. 5) similar to what was found in tumor bearing mice [1]. Given the inability for INDO to block the elevation of plasma CARTp in response to LPS, and the correlation of CARTp and SAP levels, we are left to conclude that the plasma CARTp response to LPS is prostanoid independent, but rather, driven by inflammatory signals other than those induced by cyclooxygenase.

Given genetic changes in the ARC which were found to attend LPS-induced inflammation, and that the ARC plays a role in feeding controls, it is tempting to speculate as to whether such expressional changes may contribute to the feeding response in illness. It is already well described that food intake is diminished in response to LPS, and as expected, the mice did display a significant weight loss 24 h after LPS treatment. In the healthy state, CARTp or TSH each reduces caloric intake upon intraventricular injection [9, 11, 12, 27, 42], and CART mRNA is down-regulated in food-deprived animals and up-regulated after feeding [9, 44]. In mice bearing pro-inflammatory tumors, a similar down-regulation of hypothalamic CART mRNA expression is seen [1]. Together, our results may thus infer that down-regulation of CART and TSHR mRNA in the ARC could be a compensatory suppression rather than causal of anorexia in states of inflammation, perhaps to defend body weight in states of disease.

## Conclusions

In conclusion, our findings show that expression of TSHR and of CART is suppressed in response to LPS within the ARC in the hypothalamus of the mouse. The present findings highlight hypothalamic TSH receptor mechanisms and CART as putative adaptive neuroendocrine components in the response to acute inflammation. Given the role of the ARC in feeding controls, and the observed weight loss in response to LPS treatment (Fig. 2), this may be of relevance for energy balance during states of disease. Our findings also demonstrate that plasma CARTp is induced in, and correlates to inflammation. The possible functional implication of increased circulating CARTp levels in inflammation remains to be established.

## Data Availability

All collected and analyzed data in the study is presented in the text. Raw data can be made available from the corresponding author on reasonable request.

## Abbreviations

LPS: lipopolysaccharide
CART: cocaine- and amphetamine-regulated transcript
TSH: thyrotropin
SAP: serum amyloid protein
TSHR: TSH receptor
s.c.: subcutaneous
